# Evidence for infectious merozoites of Plasmodium falciparum from natural isolates of cultured hepatoma cells infected with sporozoites

**DOI:** 10.1371/journal.pone.0319901

**Published:** 2025-03-19

**Authors:** Olumide Adeyemi, Akinniyi Osuntoki, Olubunmi Magbagbeola, Muzamil Mahdi Abdel Hamid, Arwa Elaagip, Ann-Kristin Mueller, Muntaser Ibrahim

**Affiliations:** 1 Department of Biochemistry, Faculty of Basic Medical Sciences, College of Medicine, University of Lagos, Lagos, Nigeria; 2 Department of Parasitology and Medical Entomology, Institute of Endemic Diseases, University of Khartoum, Khartoum, Sudan; 3 Heidelberg University Hospital, Department of Infectious Diseases, Heidelberg, Germany; 4 Department of Molecular Biology, Institute of Endemic Diseases, University of Khartoum, Khartoum, Sudan; Penn State Health Milton S Hershey Medical Center, UNITED STATES OF AMERICA

## Abstract

Previous cell culture systems using various human hepatoma cell lines established that the intra-hepatic stages of *Plasmodium falciparum* could be studied *ex vivo.* However, only one of these culture systems yielded infective merozoites that subsequently completed the parasite’s life cycle outside a human host. We hypothesized that a major limitation is the use of laboratory-adapted *P. falciparum* blood stages for sporozoites generation. *Plasmodium falciparum* sporozoites were generated by membrane-feeding of gametocyte-infected blood samples from hospital patients to *Anopheles arabiensis*. Subsequently, cultured HepG2 cells were infected with the sporozoites. From 6 days post-sporozoite inoculation, liver merozoites could be harvested from the cell supernatants. When co-cultured with O^ +^ erythrocytes, these merozoites established a blood infection and yielded erythrocytic stage parasites that re-infected erythrocytes. To confirm that the erythrocytic parasites generated were *P. falciparum*, RNA expressed by the erythrocytic parasites was isolated and used as control in microarray analysis against RNA expressed by irradiated erythrocytic parasites; subsequently, *P. falciparum* genes were identified. The cultured HepG2 cells permitted the full intra-hepatic maturation of *P. falciparum* parasites from natural isolates. Infective merozoites were yielded which gave rise to the erythrocytic stage *P. falciparum* post-infection into O^ +^ erythrocytes. The full intra-hepatic maturation of the naturally isolated *P. falciparum* parasites in a HepG2 cell culture system is possible. This finding has important implications for malaria research and vaccine development.

## Introduction

Malaria is a vector-borne disease caused by the apicomplexan parasite, *Plasmodium,* and considered a major global health concern. There are currently 247 million malaria cases reported in 84 malaria endemic countries; of these, 619,000 died in 2022 [1]. Infection of malaria in humans is caused by five *Plasmodium* species: *P. falciparum*, *P. vivax*, *P. ovale* (*curtisi* and *wallikeri* strains), *P. malariae* and *P. knowlesi*, with *falciparum* causing most cases [2].

The laboratory study of human malaria is associated with a host of challenges – majority of which arise from the ethical issues associated with using human subjects. Hence, the focus of biomedical research has shifted to studying parasites *ex vivo* after adaptation to blood culture or with long-term laboratory-adapted strains for *in vitro* culture systems. Several culture systems that support different *Plasmodium* species, particularly their asexual blood stage replication, have been developed [3].

The liver phase of the parasite’s life cycle however remains the most difficult to simulate in the laboratory [4]. Two landmark studies have shown that using primary human hepatocytes (PHH) allow at least partial liver stage development after inoculation with sporozoites from laboratory isolates [5,6]. Since obtaining primary liver cells from hospital patients remains challenging, different human hepatoma cell lines have been developed and applied to support the laboratory culture of human malaria parasites [7,8]. One of the cell lines, Huh-1, was reported to support the development of the *P. falciparum* liver stages beyond the uninucleate stage, but maturation into schizonts was not observed [9]. HHS-102, a Thailand-derived cell line that has morphological similarities to PHHs, has been shown to support *P. falciparum* sporozoite infection (albeit at a very low rate of 0,009%), merozoite egress and their invasion of human erythrocytes [7].

HepG2 cells were previously shown to support liver stage maturation of different strains of *P. vivax* [10,11], it was however reported that *P. falciparum* does not achieve complete maturation in HepG2 cells [8,12]. HC-04 is however reported to support the growth of both *P. falciparum* and *P. vivax* [12]. A new subclone of HC-04, HC-04J7, is reported to better support the development of *P. falciparum* in culture [13]. However, with HC-04J7, the culture medium must be changed every 24hrs; also, because this cell line cannot survive beyond 5 days in culture, it cannot support *P. vivax* hypnozoites and the *in vitro* life cycle [13].

The use of iPSC-derived hepatocyte-like cells (iHCLs) to support the *in vitro* growth of *P. falciparum* and *P. vivax* has also been reported [14]. iHCLs support *P. falciparum* sporozoite infection and development into hepatic schizonts. For *P. vivax*, hypnozoite development and the emergence of erythrocytic forms were not reported in iHCLs [14].

The use of coculture models, the most popular of which is the micropatterned coculture (MPCC) [4,15], has also been reported. MPCC is reported to support *P. falciparum* sporozoite infection, the production of schizonts, merozoite egress and the subsequent production of erythrocytic forms of the parasite [15]. With coculture models, malaria culture systems are now automated with medium throughput capacities [15,16].

Since the invasion of *P. falciparum* into HepG2 is possible, and published data only reported on the use of laboratory strains of *P. falciparum* for infection studies, we wanted to test whether the major limitation in the parasite’s *in vitro* studies, using human hepatoma cell lines, can be assigned to the transcriptional variations in the parasite rather than the host hepatoma cell.

Here, we describe our studies on complete maturation of naturally isolated *P. falciparum* inside HepG2 cells and their capacity to establish a blood infection in co-cultured erythrocytes.

## Materials and methods

### Preparation and culture of cell line

The HepG2 cell line used in this study was kindly provided by Dr. Ann-Kristin Mueller, from the Parasitology Unit, Department of Infectious Diseases, Heidelberg University School of Medicine.

The HepG2 cells’ culture and infection experiments were carried out at the molecular biology laboratory of the Institute of Endemic Diseases of the University of Khartoum, Sudan. The HepG2 cells were seeded at a density of 50,000 – 70,000/25 cm^2^ flasks in complete DMEM (Dulbecco Modified Eagle’s Medium - Sigma) (17.3g media, 3.7g/L NaHCO_3_, 10% FBS, 4µl/L insulin, 100units/ml penicillin, 100 μg/ml streptomycin, 2mM L-glutamine, 10^-6^ mM hydrocortisone, pH 6.3) and incubated at 37^°^C in an incubator using an air-tight candle jar. The culture medium was changed every 3 days until sub-confluency was reached. For subsequent sub-culture, the cells were washed with PBS, trypsinized with 0.25% trypsin at 37^°^C for 2mins and re-suspended in DMEM complete. The cells were then split at ratios that give seeding densities of 5-7 × 10^4^ and introduced into new culture flasks. The medium was changed every 3 days. To check the cells’ viability, they were stained with 0.1% trypan blue and scored using an inverted microscope. Cell passages were recorded and seed lots were stored [17, 18].

### Characterization of HepG2 cells

To determine the growth kinetics, the cells were seeded in triplicates (HepG2A, HepG2B, HepG2C) at a density of 6.5 × 10^4^ per 25 cm^2^ culture flask. The cells were then harvested at 24hr intervals for 7 days and counted using a hemocytometer. To quantify a signature hepatocyte enzyme activity, the cells’ glucose-6-phosphatase (G6Pase) specific activity was determined by incubating them with 100 μl of 0.1M glucose-6-phosphate and measuring the free phosphate released into the medium. In addition, cellular albumin and globulins secretion by the cells was investigated using SDS-PAGE. Three culture flasks containing the cells at the 20th, 21st and 22nd passages were taken. The cells were first washed out of their culture flasks before washing thrice with sterile PBS and lysed by adding 20 μl of 2 ×  SDS-PAGE sample buffer (4mMTris-HCl, pH 6.8, 10% w/v glycerol, 25w/v SDS, 5% β-mercaptoethanol, 0.05% w/v bromophenol blue). After protein denaturation at 95°C for 4mins, the resulting solutions were spun, and the protein extracts were centrifuged at 1300rpm for 30secs. Five µl of the supernatants of each flask was then taken and introduced in duplicates into the SDS-PAGE wells (using purified human blood serum as the marker). Protein separation was carried out at a constant voltage of 80V for 2hrs [17].

### Sporozoite preparation

*Anopheles arabiensis* from a susceptible laboratory strain (Dongola colony) at The Department of Medical Entomology, National Public Health Laboratories, Federal Ministry of Health, Sudan, were fed on blood containing *P. falciparum* gametocytes using the artificial membrane feeding technique [19]. At 15-18 days post-blood feeding, the mosquitoes were lightly anesthetized with chloroform and ground in 2ml dissection medium (PBS +  0.001% FCS). Sporozoites were isolated from the pulverized mosquitoes using the Sucrose Gradient Centrifugation technique [20]. The sporozoites were pooled in 200µl of DMEM medium, transferred to 1ml Eppendorf tubes and kept on ice. To detect the isolated sporozoites, 10µl of the sporozoite solution was smeared onto glass slides and fixed with methanol. The sporozoites were stained with freshly prepared Giemsa, viewed under the microscope and micrographs were taken [17].

### Infection of the HepG2 cells with P. falciparum sporozoites

The sporozoite infection experiments were carried out with HepG2 cells at passages 32-35. The log phase cells were first transferred into 24-well plates at a density of 5 × 10^4^ per well. To each well 5 × 10^4^ of the freshly isolated sporozoites were added. The cells were incubated at 37^°^C and medium changed every 3 days [17].

### Co-culture with erythrocytes, merozoite invasion and culture of erythrocytic parasites

Five ml of human O^ +^ blood samples were collected from volunteers with negative Giemsa results on 17/02/2011, 24/02/2011, and 03/03/2011. The blood samples were washed three times with an equal volume of normal saline and added to each well at 2% hematocrit 3, 5, 7 and 9 days post-sporozoite inoculation. Five µl of the RBCs was then taken from each well 48, 96 and 144hrs post-erythrocyte addition and used to prepare blood smears. The dried smears were stained with Giemsa and examined for asexual blood stage parasites [17].

### Erythrocyte harvest and cryopreservation

For wells with positive Giemsa results, their RBC contents were taken out and spun down at 3000rpm for 15mins. The pellets obtained were introduced into cryovials into which 1ml of RNAlater® (Qiagen, USA) was added. These were mixed and stored at -80^°^C for subsequent gene expression analysis.

### RNA isolation and microarray identification of *P. falciparum* genes in erythrocytic parasites

To confirm that the parasites seen in the blood pictures are truly *P. falciparum* (and not another organism) [21,22], a microarray study was carried out (this procedure was also carried out as part of a larger study where part of the sporozoites generated were attenuated using 10Krad dose of x-irradiation to produce radiation attenuated sporozoites (RAS) [17]). Subsequently, the two sporozoite groups (RAS and Control) were used to infect the human hepatoma cell line, HepG2, to yield merozoites and then erythrocytic stage parasites. Total RNA isolation was carried out on the infected erythrocytes in both groups followed by genome-wide expression profiling.

Using the manufacturer’s protocol, the RNeasy mini kit (Qiagen, USA) was used for total RNA isolation from cryovials containing the infected RBCs. The final volume of the elution was 40µl. Aliquots of the isolated RNA were added to the NanoDrop ND-1000 Spectrophotometer (Peqlab, Germany) and the RNA’s concentration and purity were determined. RNA Integrity values (RIN) were obtained for each of the RNA samples using the 2100 Bioanalyzer (Agilent Technologies, USA). RNA samples with RIN ≥  7 were used for microarray hybridization and gene expression analysis. Using the online tool from eArray (http://earray.chem.agilent.com/AgilentTechnologies) an Agilent Custom *P. falciparum* Gene Expression Microarray (8 ×  15K format, AMADID 040812, Agilent Technologies) was designed for this analysis. Probe information regarding the coding sequences of the relevant *P. falciparum* 3D7 transcriptome was retrieved from the NCBI reference sequence of the assembly ASM276v1 (http://www.ncbi.nlm.nih.gov/assembly/360518). The isolated total RNAs were spiked with *in vitro* synthesized polyadenylated transcripts (One-color RNA Spike-in Mix, Agilent) transcribed into cDNA and converted into labeled cRNA by *in vitro* transcription (Quick-Amp Labeling kit, One-Color, Agilent) incorporating Cyanine-3-CTP. Each of the labeled cRNA (0.60µg) was then hybridized at 65^°^C for 17hrs on the custom *P. falciparum* gene expression microarray. Quantile normalization was carried out on the raw signal intensities of each array using the software, GeneSpring GX12 (Agilent). Welch’s approximate t-test was used to compare the genes expressed by the erythrocytic parasites and those expressed by sample irradiated erythrocytic parasites (used in a larger study [17]); this gave *p*-values for each gene detected on the array.

### Ethical approval and consent to participate

The study protocol was reviewed and received ethical approval from the Research Ethics Committee of the Institute of Endemic Diseases, University of Khartoum (Ref No. UKIENDER100901).

Patients hospitalized for treatment of *P. falciparum* malaria at Khartoum Paediatric Hospital and the Khartoum General Hospital were recruited to participate in this study from January to March 2011. A written informed consent was obtained from all participants who were aged 18 years old and above. For children between 5 and 13 years, the consent of their parents or legal guardians was sought. For children between 13 and 18 years, assent from the child and consents from the parents or legal guardians were obtained. After this, routine blood samples obtained from the patients were screened for the presence of *P. falciparum* ring stage and gametocytes. Five ml blood was withdrawn by venous puncture from 10 patients with parasitemia between 1.5 - 2.2%.

## Results

### Characterization of cultured HepG2 hepatoma cells

**Fig 1:**
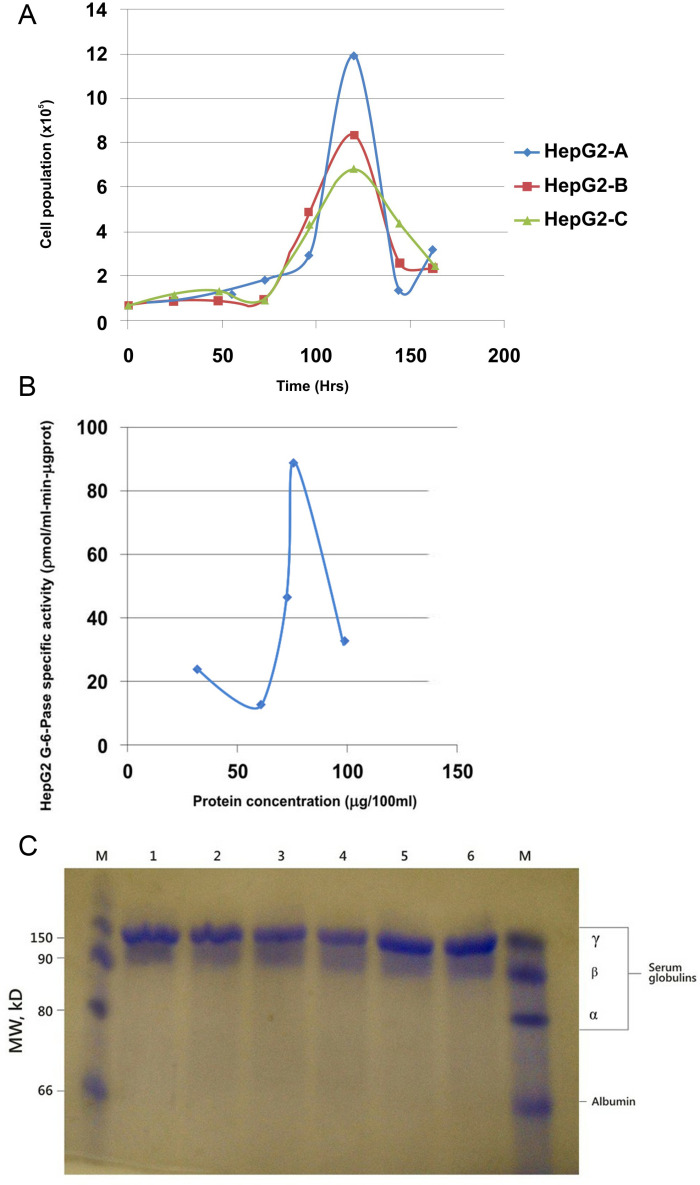
A) HepG2 cells’ growth kinetics in DMEM complete media. Cell population was highest on day 5 (120hrs) reaching a peak of 11.825 ×  105 cells/25 cm^2^ flask – an 18.2-fold population increase from 0-hr. **B) Saturation curve for the HepG2 G-6-Pase specific activity with increasing concentrations of HepG2 protein.** The peak G6Pase specific activity before enzyme’s active sites were completely saturated was 9.1ηmol/min-mgprot. **C) SDS-PAGE result for the HepG2 cell line’s secreted proteins.** Lane M (Control) shows the serum proteins, albumins, and globulins. Lanes 1-6, showing the proteins synthesized and stored in the cells’ cytosol, give a similar pattern for the cells from the three flasks. γ-globulins were detected while albumin, α−and β−globulins secretions were not seen.

The growth curves of the HepG2 cells were monitored (Fig 1A). Analysis of the growth curve showed that at a starting population of 6.5 × 10^4^, the mean doubling time was approximately 48hrs. At the log phase, the population increased exponentially until 120hrs (day 5) when it had increased approximately 18-fold.

Then, the G6Pase specific activity was measured, as a functional read-out for gluconeogenesis in the cultured cells (Fig 1B). The saturation curve for G6Pase specific activity with increasing HepG2 protein concentration showed that the enzyme’s specific activity increased gradually with increasing HepG2 protein/enzyme concentration. The G6Pase concentration became the limiting factor when the enzyme’s level matched the concentration of the available substrate. The highest G6Pase specific activity for the cells was 9.1ηmol/min-mgprot.

The proteins expressed by the HepG2 cells were detected using SDS-PAGE (Fig 1C). On the control lanes M (Marker), four clusters of proteins, representing the major human serum constituents can be seen: albumin and globulin fractions (α, β, and γ – globulins). The HepG2 cells (lanes 1-6) have about the same protein pattern – albumin, α− and β-globulin secretions were not detectable in them. In contrast, γ−globulins secretions were detected across the six lanes.

### Sporozoite generation and HepG2 cell infection

**Fig 2:**
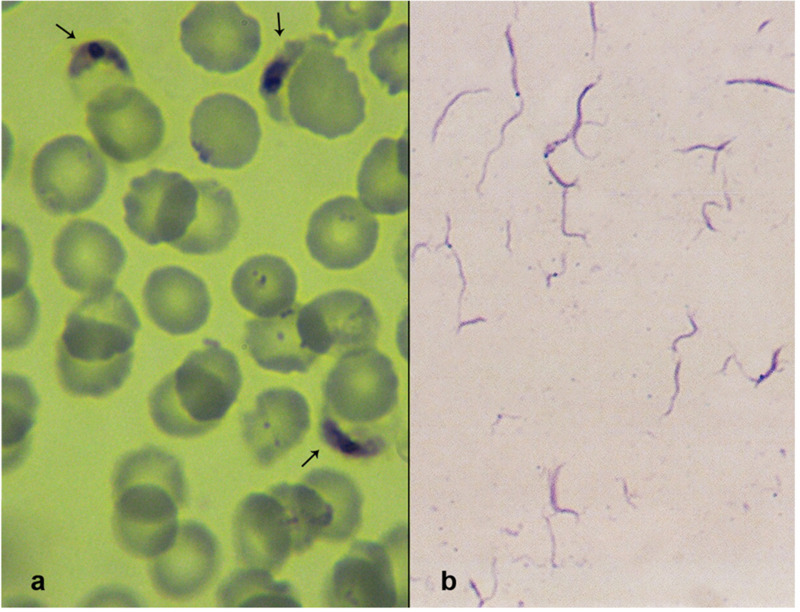
A) *Plasmodium falciparum* gametocytes from blood samples obtained from hospital patients in Khartoum, Sudan. B) *P. falciparum* sporozoites isolated from the infected *Anopheles arabiensis* mosquitoes 15-18 days post-gametocyte blood feeding.

Successful sporozoite production was monitored in infected *An. arabiensis* mosquitoes. The isolated *P. falciparum* sporozoites were detected by Giemsa staining (Fig 2). The sporozoites were long and slender with fine structures and pointed ends. Generally, their diameters were uniform; some however were enlarged in the middle due to their characteristic central and elongated chromatin. From three successful infections, approximately 3.6 × 10^6^ sporozoites were obtained.

### Detection of merozoites from cell culture supernatants

Merozoites were detected in the wells from day 6 onwards post-sporozoite inoculations. From the 21 positive wells, approximately 9.11 ×  10^4^ infective merozoites were obtained.

**Fig 3:**
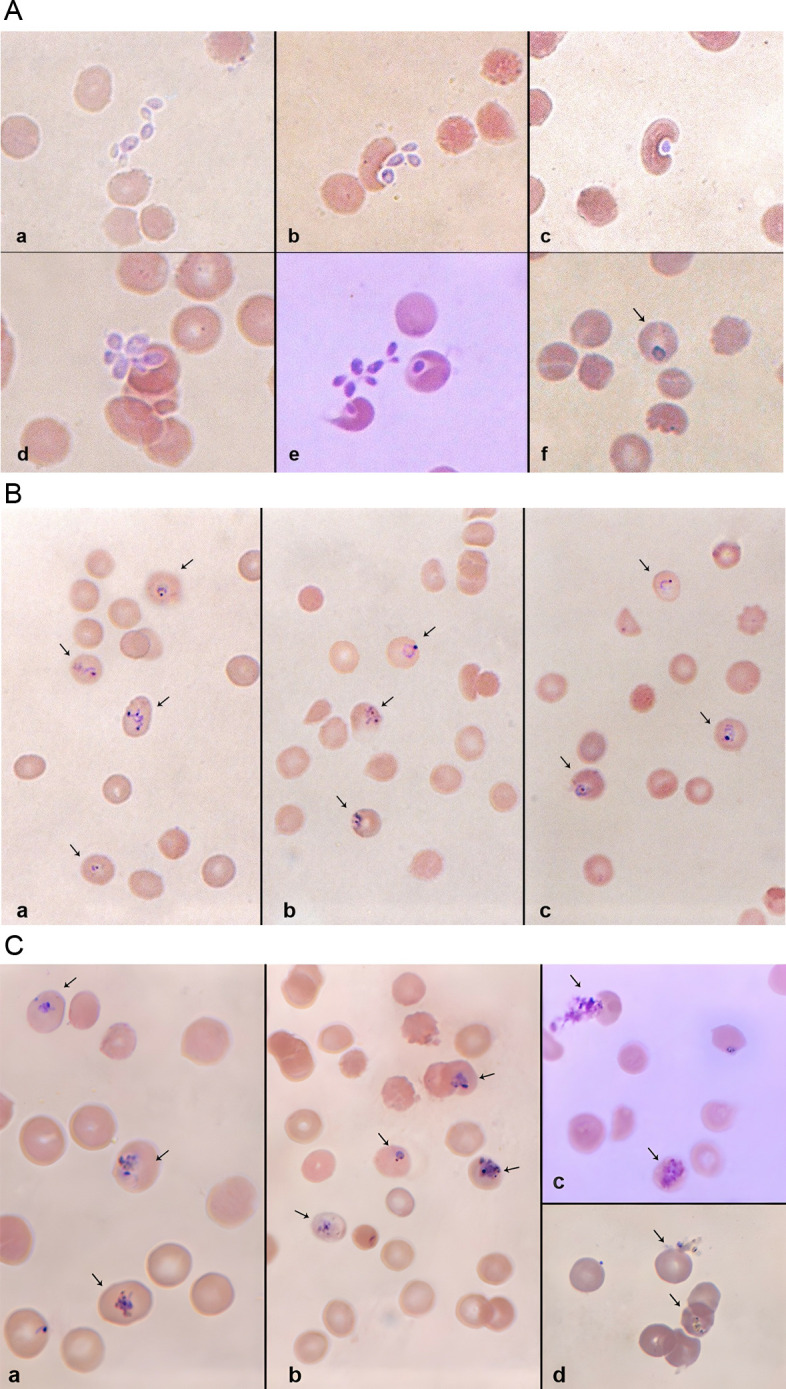
A) Extracellular parasites, merozoites that emerged from the infected HepG2 cells 6 days post-sporozoite inoculation. The Giemsa-stained films detected the entire sequence of the parasite’s infection into the erythrocytes. (a) Shows the merozoites locating the erythrocytes. (b) Shows the initial recognition events, the subsequent attachment to the erythrocyte membrane and junction formation on the red cells. (c-d) Shows the erythrocyte’s invagination and engulfing of the merozoite; multiple infection of the erythrocytes (unique to *P. falciparum*) can also be seen. (e-f) Shows the merozoite now fully internalized into the erythrocyte. B) Giemsa-stained micrographs of ring-stage Plasmodium falciparum detected 48, 96 and 144hrs post-erythrocyte addition. Parasites have a defined chromatin dot with a bluish cytoplasm. Note the multiply infected erythrocytes in (a) and (b). C) Giemsa-stained micrographs of Plasmodium *falciparum trophozoites* and schizonts detected 48, 96 and 144h post-erythrocyte addition. (a and b) Trophozoites’ cytoplasms are denser and their ring shapes have gradually given way to a more amoeboid structure. (c) A ruptured schizont and a developing one. (d) Another ruptured schizont and a developing one.

The entire sequence of merozoite infection of erythrocytes, that is, initial recognition, attachment, junction formation and entry could be observed after O^** +**^ erythrocytes were added to the wells (Fig 3A). When the infected erythrocytes were cultured for an additional 48hrs, the parasite’s erythrocytic stages could be detected in Giemsa-stained blood films. With a second and third round of erythrocyte addition, more erythrocytic forms were detected (Fig 3B and 3C). The percentage of parasitemia on days 7, 9 and 11 for the three successful *in vitro* cultures are shown in Table 1.

**Table 1:** Mean percentage parasitemia of erythrocytic stage *Plasmodium falciparum* in three culture samples * .

Groups	Mean Percentage Parasitemia (Mean ± SEM*)		
Day 7	Day 9	Day 11
Group1 (n = 9)	0.061 ± 0.014	0.307 ± 0.098	1.180 ± 0.250
Group 2 (n = 8)	0.000	0.268 ± 0.053	1.110 ± 0.270
Group 3 (n = 10)	0.000	0.366 ± 0.041	1.707 ± 0.205

* On day 7, erythrocytic samples were observed in Group 1, 48hrs after erythrocyte addition; erythrocyte stage parasites were observed in the other two groups on days 9 and 11 (48 hrs after a second and third erythrocyte addition). The highest mean percentage parasitemia on day 11 was observed in Group 3, showing a higher parasite multiplication compared to the other two samples.

* SEM is Standard error of the mean.

### Identification of *P. falciparum* genes in erythrocytic parasites

The differentially expressed genes on the Agilent Custom *P. falciparum* microarray after hybridization of the sample RNA from the RAS and Control parasites are shown on Table 2. Several *P. falciparum* marker genes are seen, including the three genes the organism uses for antigenic variation, *var*, *rif* and *stevor*.

**Table 2:** Genes of *Plasmodium falciparum* identified by microarray in RNA expressed by the erythrocytic parasites that developed after merozoites co-culture with erythrocytes.

S/N	Identified Genes	Gene Symbol	P-value
1	Ribonuclease HII, putative (*P. falciparum* 3D7)	PFF1150w	0.028
2	60S Ribosomal protein L10, putative (*P. falciparum* 3D7)	PF14_0141	0.045
3	Regulator of chromosome condensation, putative (*P. falciparum* 3D7)	PF13_0303	0.013
4	Thymidylate kinase, putative (*P. falciparum* 3D7)	Tmk	1.88X10^-4^
5	Rhoptry neck protein 2 (*P. falciparum* 3D7)	PfRON2	0.006
6	Conserved *Plasmodium* protein (*P. falciparum* 3D7)	PF10_0029	0.028
7	Replication factor C, subunit 2 (*P. falciparum* 3D7)	PFB0840w	0.036
8	Rifin	PF10_0002	3.88X10^-4^
9	Histone deacetylase, putative (*P. falciparum* 3D7)	PF10_0078	0.015
10	Ctr Copper transporter domain containing protein, putative (*P. falciparum* 3D7)	PF14_0211	0.011
11	Ethanolamine kinase, putative (*P. falciparum* 3D7)	PF11_0257	6.64X10^-4^
12	Asparagine-rich antigen	PF08_0060	0.009
13	MAC/Perforin putative (*P. falciparum* 3D7)	PFD0430c	0.001
14	Coronin binding protein (*P. falciparum* 3D7)	PFF1110c	0.041
15	Histone deacetylase putative	PF10_0078	0.012
16	Protein kinase putative (*P. falciparum* 3D7)		8.48X10^-4^
17	Rifin	PFL2655w	0.048
18	60S Ribosomal protein L37ae	PFB0455w	0.013
19	Rifin (*P. falciparum* 3D7)	PF10_0003	0.035
20	Erythrocyte membrane protein1 (PfEMP1) –like protein (*P. falciparum* 3D7)	VAR-like	0.003
21	Merozoite surface protein 10, MSP 10 (*P. falciparum* 3D7)	PFF0995c	0.002
22	Rifin	PFL0105w	0.002
23	Rifin	PF10_0401	4.86X10^-5^
24	Iron-sulfur assembly protein, putative (*P. falciparum* 3D7)	PFB0320c	0.042
25	CPW-WPC family protein (*P. falciparum* 3D7)		0.002
26	Replication factor C subunit 5, putative (*P. falciparum* 3D7)	PF11_0117	0.042
27	Putative cytochrome oxidase III (*P. falciparum* 3D7)	coxIII	0.036
28	Stevor (3D7-StevorT3-2) (*P. falciparum* 3D7)	MAL3P7.52	0.006
29	Serine/Threonine kinase -1, PfLammer (*P. falciparum* 3D7)	LAMMER	0.001
30	Merozoite surface protein 10, MSP10 (*P. falciparum* 3D7)	PFF0995c	0.013
31	28KDa Ookinete surface protein (*P. falciparum* 3D7)	PF10_0302	0.003
32	Heat shock protein DnaJ homologue Pfj2 (*P. falciparum* 3D7)	PF11_0099	0.013
33	Ubiquitin conjugating enzyme, putative (*P. falciparum* 3D7)	PFI1030c	0.001
34	Early transcribed membrane protein 4, ETRAMP4 (*P. falciparum* 3D7)	PFD1120c	0.021
35	Var (3D7-varT3-2) (*P. falciparum* 3D7)	PF3D7VART32	0.005
36	Translation-initiation factor-like protein (*P. falciparum* 3D7)	PF08_0018	0.015
37	Heme-detoxification protein (*P. falciparum* 3D7)	HDP	0.032
38	Formin2, putative (*P. falciparum* 3D7)	PFL0925w	0.045
39	GDP-dissociation inhibitor domain containing protein (*P. falciparum* 3D7)	PF10_0373	0.005
40	Flap endonuclease1 (*P. falciparum* 3D7)	PFD0420c	0.007
41	*Plasmodium* exported protein (PHISTc) (*P. falciparum* 3D7)	PFB0905c	0.001
42	DNA Polymerase epsilon subunit B, putative (*P. falciparum* 3D7)	MAL3P3.4	0.001
43	Transcription factor with AP2 domain(s), putative (*P. falciparum* 3D7)		0.002
44	Stevor (*P. falciparum* 3D7)	PFD0035c	0.005
45	Clathrin-adaptor medium chain, putative (*P. falciparum* 3D7)	PF13_0062	0.018
46	Cyclophilin, putative (*P. falciparum* 3D7)	PF14_0223	0.009
47	Mitochondrial phosphate carrier protein (*P. falciparum* 3D7)	mpc	6.86X10^-6^
48	Outer arm dynein Ic3, putative (*P. falciparum* 3D7)		0.046
49	Kelch protein, putative (*P. falciparum* 3D7)	MAL7P1.137	0.002
50	Erythrocyte Membrane Protein 1 (PfEMP1), truncated (*P. falciparum* 3D7)	PFB0020c	0.049
51	Phospholipase A2, putative (*P. falciparum* 3D7)	PFB0410c	0.009
52	Anamorsin related protein, putative (*P. falciparum* 3D7)		0.021
53	Rac-betaserine/threonine protein kinase, PfPKB (*P. falciparum* 3D7)	PKB	0.031
54	Sortilin, putative (*P. falciparum* 3D7)	PF14_0493	0.005
55	Chromatin assembly factor 1 P55 subunit, putative (*P. falciparum* 3D7)	PF14_0314	0.036
56	3D7 Surf1.3;Surface-associated interspersed protein 1.3 (SURFIN1.3) (*P. falciparum* 3D7)	PFA0725w	0.021
57	Asparagine-rich protein (*P. falciparum* 3D7)	PFD1105w	0.004
58	Erythrocyte Membrane Protein 1 (PfEMP1) (*P. falciparum* 3D7)		0.01
59	Splicing factor, putative (*P. falciparum* 3D7)	MAL13P1.120	0.045
60	Surface-associated interspersed gene 8.3 (SURFIN 8.3) (*P. falciparum* 3D7)		0.014
61	GPI8p transamidase (*P. falciparum* 3D7)	PF11_0298	0.009
62	Mitochondrial ribosomal protein L2 precursor (*P. falciparum* 3D7)	PF11_0337	0.036

## Discussion

Since Trager and Jensen (1976) [23] developed the *in vitro* methods of *Plasmodium* cultivation, much progress has been made in the study of the parasite and in the management of the disease. Though most of the study initially focused on the parasite’s blood stage forms, research into the hepatic stages has also been undertaken using different forms of hepatocyte cultures. Following its isolation and characterization in 1975 [24], HepG2 has been used severally in many *in vitro* studies of different *Plasmodium* species, including *falciparum* and *vivax*. The choice of HepG2 for many of these studies is largely due to the fact that many of the normal hepatocyte functions have been found to be conserved in it [25]; though chromosomally abnormal [26], many liver-specific genes are still expressed by the cell line [25] with several biochemical and metabolic characteristics of normal human hepatocytes maintained [27].

Although all HepG2 cell lines obtainable in research laboratories across the world are derived from the same parent cell line [24,28], given the slightly different *in vitro* conditions the cells are subjected to, alongside the prolonged time of culturing in these laboratories, phenotypic variations in sub-lines of HepG2 from various laboratories are often observed [29]. This is evident on comparing the assay results of the HepG2 used in this study to that of other HepG2 in literature. A HepG2 sub-line cultured in a laboratory at the Imperial College London showed cell growth kinetics parameters of 33.44hrs lag phase, 93hrs log phase and a population doubling time (PDT) of 43.92hrs [28]. The HepG2 cells used in this study, obtained from Heidelberg Germany, gave a 72hrs lag phase, 48hrs log phase and a PDT of 87.2hrs as its cell growth parameters. Hence, though these two cell lines originally came from the same parent stock, the variation in their cell growth patterns is evident. The Heidelberg line used in this work uses approximately twice as much time for its lag phase and PDT as the London line uses. Also, the Heidelberg line uses about half as much time in the log phase as the London line. Such variation is also observable in the glucose-6-phosphatase activities of the HepG2 sub-lines; while Jetsumon et al., (2006) [12] reported a HepG2 glucose-6-phosphatase specific activity of 6.38µmol/min-mg-prot, the Heidelberg HepG2 sub-line used in this study showed a much higher enzyme specific activity of 9.06µmol/min-mg-prot.

The phenotypic variations seen in the sub-lines of hepatocytes are known to affect how susceptible they are to *Plasmodium* sporozoite entry when used for infection studies in the laboratory [28]. Though HepG2 cells are known to support the complete intrahepatic growth of *Plasmodium* species like *P. berghei* [30] and *P. vivax* [11], subsequently yielding infective merozoites, *P. falciparum* is not known to achieve full maturation in any HepG2 cell subline [8,12]; neither are infective merozoites yielded [12]. In this study, however, a different scenario was observed; using wild-type *P. falciparum* sporozoites, a complete intrahepatic growth and the subsequent release of merozoites was seen with the HepG2 cell line obtained from Heidelberg. Several cases of *P. falciparum* co-infections with organisms like *Clostridium perfringens* and *Candida spp.* [22] and *Candida albicans* [31] in patients have been reported. We reasoned that since the O^ +^ blood samples collected from volunteers in this study were only screened for malaria parasites (yielding negative Giemsa results), it was also necessary to screen the blood stage parasites obtained after the sporozoite-infection and erythrocyte co-culture experiments in this study for *P. falciparum* specific genes. This became even more necessary when we consider that peripheral blood smears of *Candida albicans* resemble the Giemsa blood smears of *P. falciparum* merozoites [21].

From the microarray results in this study, several *P. falciparum* marker genes were detected, including the important *P. falciparum* virulence determinant genes, *var*, *rif* and *stevor* [32], confirming the identity of the parasite seen in the blood smears as *P. falciparum*.

The success of the HepG2 sub-line used in this work in supporting wild-type *P. falciparum* sporozoites’ full intrahepatic growth brings to the fore some important questions concerning the inability of other HepG2 sub-lines (and indeed other characterized human hepatoma cell lines) to fully support this important biological process in the *P. falciparum* life cycle. Is phenotypic shift, owing to the host laboratory’s *in vitro* conditions, accountable for the successful exo-erythrocytic processes of the wild-type parasite in the HepG2 cells used in this study? Or, has the major limitation for the successful maturation of the parasite in HepG2 cell line been the continued use of laboratory-adapted *P. falciparum* [33] erythrocytic stages for the generation of sporozoites? Or is it a combination of both factors that made the parasites’ full exo-erythrocytic maturation possible in this study?

Studies have shown that there are significant transcriptional differences between natural isolates and laboratory-adapted strains of *P. falciparum* [34]. Much of the biological variations observed occurred because of the loss of small or large chromosomal segments in the laboratory-adapted strains [35]. This also occurs because of gene copy number variations in the laboratory strains’ genome [35]. It is believed that the parasite maintains these variations because of the conditions of its environment [34]. The *in vitro* conditions that laboratory-adapted *P. falciparum* [33] are subjected to do not present the immune challenges seen when the parasite is in the human host; thus, the laboratory-adapted parasite does not encounter the many physiological constraints the wild-type parasite is exposed to. Hence, most laboratory-adapted *P. falciparum* have either down-regulated or completely deleted segments of their chromosomes that code for factors which ensure survival when in their native host environment [36]. This explains why usually, several energetically demanding (yet crucial *in vivo*) processes, like maintaining antigenic variations and gametocytogenesis, are not observed in laboratory-adapted *P. falciparum* [35,36]. This could also account for why these laboratory-adapted parasite strains have lost the capacity for complete intra-hepatic maturation in HepG2. The ability of the wild-type parasite to achieve this in the HepG2 (Heidelberg) cell line used in this study strongly alludes to this. Further infection studies where the wild-type parasite is transfected into different HepG2 sub-lines that have been cultured in other laboratories would help to verify this important observation. Also, this procedure can be replicated using other established human hepatoma cell lines that have hitherto been refractive to *P. falciparum*’s *in vitro* culture.

A major limitation of human malaria research and what should be the resultant identification of candidate genes for the effective malaria vaccine development is the unavailability of sufficient *in vitro* systems that support the parasite’s intrahepatic development. The finding of this study helps add at least one more cell line to the list of hepatoma cell lines that support *P. falciparum in vitro* growth; furthermore, since HepG2 is a ubiquitous and relatively inexpensive hepatoma cell line, more research laboratories (especially those in developing countries where malaria is endemic) can now set up viable *in vitro* malaria research systems to further study this disease. This could ultimately have very important implications for human malaria drug and vaccine development.

## Conclusions

The findings of this study, where the infective merozoites and the erythrocytic stages were produced when the natural (wild-type) isolate of *P. falciparum* was infected into HepG2 cells, contrast with earlier reports of the unsuitability of HepG2 for *P. falciparum* infection studies. The findings also suggest that the outcome of *Plasmodium in vitro* culture may depend more on the parasite type used than the host hepatoma cell line. This may be a result of the gene deletions and other forms of copy number variations that exist in laboratory-adapted *Plasmodia* which hampers their ability to undergo complete metamorphosis when used for *in vitro* malaria studies. However, using natural isolates of *Plasmodium* for infection studies on many of the already established human hepatoma cell lines could give a range of results that would be different from what has so far been obtained with the laboratory strains. Such results will also yield inherently greater information on the parasite’s *in vivo* existence and physiology and on the long run, positively impact malaria chemotherapeutic and vaccine development.
